# Enhancement of biomethane potential of brown sludge by pre-treatment using vortex based hydrodynamic cavitation

**DOI:** 10.1016/j.heliyon.2023.e18345

**Published:** 2023-07-16

**Authors:** Md Saiful Islam, Vivek V. Ranade

**Affiliations:** Bernal Institute, University of Limerick, Limerick, V94 T9PX, Ireland

**Keywords:** Dairy sludge, Valorisation, Vortex diode, BMP, Anaerobic digestion

## Abstract

Novel, non-thermal and economically benign pre-treatment process was developed for enhancing valorisation potential of brown sludge generated by dairy industry wastewater treatment plant (WWTP). Vortex-based hydrodynamic cavitation (HC) device was used to quantify influence of pretreatment by measuring biomethane potential (BMP) of untreated and treated brown sludge. Pre-treatment parameters, primarily, pressure drop and number of passes through the cavitation device were varied to quantify influence on BMP. BMP tests were performed at 39 °C containing 5% of total solids in each reactors using an automatic BMP measurement system containing 15 reactors with each volume of 500 mL fitted with overhead stirrer. HC treatment increased the soluble chemical oxygen demand (sCOD) by more than 25% which increased the BMP. HC treatment was able to push the BMP of treated sludge to more than 80% of the theoretical BMP. Volatile solids (VS) removal was more than 65%. Highest methane yield was 376 mL/g-VS of sludge. The methodology and results presented here show significant potential to valorise brown dairy sludge via vortex based hydrodynamic cavitation.

## 1 Introduction

Dairy processing industries are producing a large volume of sludge from raw effluents, precipitation by chemical treatment and biological treatment [1]. The properties of these waste streams mainly depend on the composition of influents and technology of the treatment process [2]. Indeed, following treatment in wastewater treatment plant (WWTP), 128,600 tons of sludge was generated in a year with the estimated 50% increase in 5 years from Irish dairy industries [3]. These wastes, containing high organics is a concern because of strict waste disposal rules to protect the environment.

After appropriate treatment, the waste sludge is disposed via land spreading or composting. It is necessary to meet the National regulations in Ireland for disposal of the sludge. The steps involved in the sludge treatment for volume and volatile matter reduction before land disposal are stabilization and thickening [1]. The sludge is either stabilized via aerobic stabilization or anaerobic digestion, activated sludge with lower concentration is accomplished in dairy waste treatment plants. For higher concentration of activated sludge, additional oxygen is required for aerobic stabilization. Unlike aerobic treatment, anaerobic digestion offers an attractive valorisation pathway. The brown sludge comprises significant carbon content and can be used as substrate in anaerobic digestion though fat in the sludge is not easily biodegradable [1,4,5]. Anaerobic digestion (AD) disintegrates organic materials in sludge [6] and produces biomethane. Fats, oils and grease (FOG) present in dairy waste streams is the challenge for bioconversion using microorganisms in AD. An appropriate pre-treatment is therefore necessary to facilitate digestion of dairy sludge in AD.

Pretreatment is used to enhance digestibility and thereby increase biogas yield. There are numerous techniques of pretreatment of biomass like physical, chemical, biological and physicochemical to make the substrate bioavailable for microbial reaction [7,8]. An attractive pretreatment process must be cost-effective, scalable, environmentally friendly and sustainable. Maximum pretreatment cost can be 40% of total cost involved in the process [9]. Physical pretreatment is used for particle size reduction for solid and dried biomass. Chemical pretreatment produced inhibitors with the severity of treatment which inhibit the microbial reaction and would involve other chemicals and costs to neutralise the treated slurry before adding into AD reaction [10,11]. Biological pretreatment is usually slow and associated cost is high [12]. Physicochemical pretreatment is relatively faster, energy efficient and less chances to produce inhibitors [13]. One of the physicochemical pretreatments is steam explosion, which requires higher energy consumption to increase the temperature and corresponding anaerobic biodegradability enhancement is not significant [14]. Acoustic cavitation and microwave have also been used for pre-treatment. However, these processes require significant energy input with adverse impact on cost and environment [15,16]. Hydrodynamic cavitation (HC) refers to a process in which small bubbles or cavities form, expand and subsequently collapse rapidly under ambient conditions. This phenomenon creates intense localized shear, hotspots and hydroxyl (OH) radicals through the decomposition of water. The intense shear, hydroxyl radicals and shockwaves generated via collapsing cavities are responsible for breakdown of particles as well as polymers. The enhanced surface area of particles because of breakage and enhanced solid-liquid mass transfer leads to significant increase in the soluble COD [17].

HC treatment is being increasingly used for the pre-treatment of biomass and different designs of HC technology are used including vortex-based cavitation devices, orifice, venturi and rotor-stator based cavitation devices [[18], [19], [20]]. Orifice/venturi-based HC device is susceptible to erosion and clogging on the other hand rotor-stator based device containing moving parts is expensive [20]. However, vortex-based HC technology is energy efficient with no risks of clogging, less chances of erosion because of cavity collapse away from wall, environment friendly and scalable pretreatment method, which has been applied effectively for degradation of dairy sludge and maximize the conversion to biomethane [10]. HC treatment of dairy processing sludge enhanced the degree of disintegration by increasing the soluble COD (sCOD) [21]. Hence, the bioavailability of the substrate increased the biomethane using microbial consortia in anaerobic digestion process.

In this work, we develop and apply HC pretreatment for brown sludge generated in dairy industry. The aim is not only to valorise brown sludge by using energy efficient and environmentally friendly HC pre-treatment but also to underpin robust, future-proofed regulatory compliance in the context of forthcoming Best Available Techniques [22]. Specific goal is to develop novel vortex-based hydrodynamic cavitation (HC) based pre-treatment of dairy sludges for better degradation of substrate enhancing soluble chemical oxygen demand (sCOD) and hence improve the bioavailability to increase the yield of biomethane.

## 2 Methodology

### 2.1 Collection of substrate and inoculum

Brown sludge samples were collected from WWTP/effluent treatment plants of local dairy industries in Ireland. Effluent treatment plants are using different wastes comes from different dairy processing steps and hence the quality of dairy processing sludge is also varied. Two types of brown sludge were taken with the different physicochemical characteristics. Inoculum was collected from a big anaerobic digester where dairy wastes are using as a substrate.

### 2.2 Characterization of brown sludge

The characterization results of brown sludge samples are shown in Table 1. The sludge samples were viscous and with high total COD content were 135.97 and 117.12 g/L for sample-1 and sample-2 respectively, where the soluble COD were 31.02 and 23.81 g/L respectively. TS content were 16.78% and 15.05% and VS content were 12.41% and 11.44% respectively for sample-1 and sample-2. Characteristics of dairy sludge with the similar properties also reported by Refs. [[23], [24], [25], [26]]. Both the samples contained very high phosphorous 5.74 and 5.45 g/L respectively. Elemental composition, CHNSO results reported in Table 1 was used to determine the theoretical BMP of the substrates.

**Table 1:** Characterization of brown sludge.

Test parameters	Sample-1	Sample-2
tCOD, g/L	135.97 ± 5.04	117.12 ± 3.07
sCOD, g/L	31.02 ± 1.01	23.81 ± 0.08
Total solid, % (w/w)	16.78 ± 0.09	15.05 ± 0.07
Volatile solids, % (w/w)	12.41 ± 0.02	11.44 ± 0.03
Total phosphorus, g/L	5.74 ± 0.02	5.45 ± 0.01
Organic acids, g/L	2.37 ± 0.01	1.78 ± 0.01
C, % (w/w)	37.46 ± 0.04	31.60 ± 0.07
H, % (w/w)	4.51 ± 0.04	4.12 ± 0.04
N, % (w/w)	7.01 ± 0.01	7.21 ± 0.02
S, % (w/w)	1.21 ± 0.02	1.74 ± 0.02
O, % (w/w)	23.57 ± 0.09	25.69 ± 0.08

### 2.3 Hydrodynamic cavitation pretreatment of Brown sludge

**Fig. 1:**
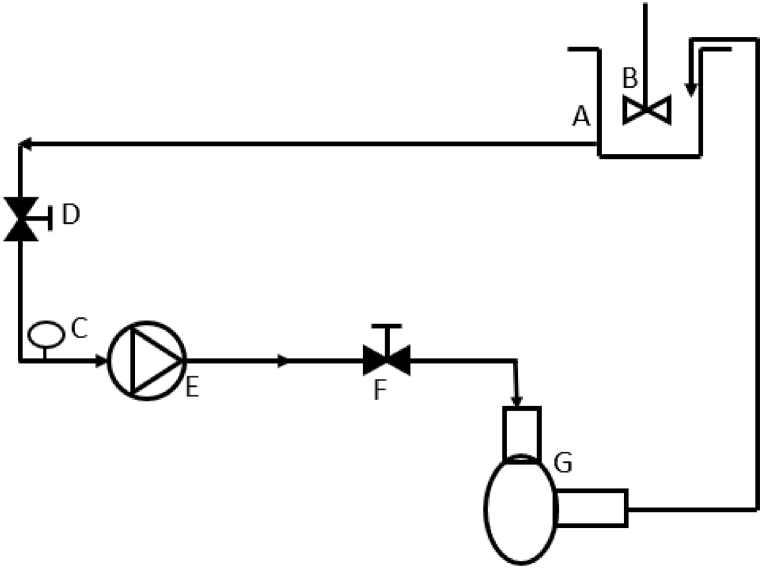
Lab scale hydrodynamic cavitation setup (A sample tank, B stirrer, C pressure gauge, D & F control valve, E pump and G vortex based HC device).

Laboratory scale hydrodynamic cavitation (HC) containing vortex based cavitation device with nominal flow rate of 1.2 LPM and centrifugal pump were used in this pretreatment process. Fig. 1 shows the schematic diagram laboratory scale HC set up. The vortex based HC device used in this work was of stainless-steel and was procured from Vivira Process Technologies. 2.5 L of substrate containing 5% of total solids (TS) (including the internal volume of pump and pipes) was treated. Substrate was diluted to that concentration by tap water and for all the dilution and BMP experiment tap water was used. The pre-treatment of brown sludge was carried out at pressure drop across HC device as 250 kPa. Samples of pre-treated sludge were collected after different number of passes through HC device (10, 20, 40 and 80 passes). All the treated and untreated samples were then characterised and used for the BMP experiment to quantify the biomethane production.

### 2.4 Analytical methods

Rheological behaviour of collected brown sludge with and without HC treatment was determined using Brookfield Ametek DV3T Rheometer [27]. Concentration of the substrate was 5% TS to measure shear strain and shear stress with different speed of spindle (0.01–60 rpm). Scanning electron microscope (SEM, Carl-Zeiss, Germany) images has been taken to check the effect of HC treatment on the substrate [28]. Washed and dried samples of both non-cavitated and cavitated samples with the different number of passes has been used to observe the morphology change due to the cavitation effect.

Total solids (TS) and volatile solids (VS) of all the substrates and inoculum used in the BMP experiment was determined according to the standard methods [29]. Total COD (tCOD) and soluble COD (sCOD), total phosphorus and organic acids were determined using the methods LCK014, LCK350 and LCK365 cuvette tests respectively (Hach-Lange, Germany). DR1900 spectrophotometer (Hach) was used for all the sample analysis. Elemental composition (CHNSO) of the sludge samples were determined by CHNSO elemental analyser.

Degree of disintegration of all the HC treated sludge were calculated according to Eq. (1) [30].

(1) $$Degree\;of\;disintegration\;DD=\frac{sCODf-sCODi}{COD-sCODi}$$

where, COD, sCOD_f_ and sCOD_i_ are the total COD of substrate, soluble COD after treatment and soluble COD without treatment in (mg/L) respectively.

### 2.5 Biochemical methane potential (BMP) measurements

The BMP measurements of untreated and HC treated samples were done using automatic methane potential test system (AMPTS) II from Bioprocess Control (Lund, Sweden). In this system there were 15 reactors with the volume of 500 mL with the working volume of 400 mL. All reactors were placed in a water bath maintaining the temperature of 39 °C. Substrate to inoculum ratio was taken at 1:2 in the reactor in terms of VS [31,32]. Each reactor was fitted with overhead stirrer and connected with the gas fixation unit by Tygon tube and connector. Continuous stirring was used with 80 rotation per minute. Produced gas from each reactor were passed through a bottle containing 80 mL of 3 M NaOH to absorb CO_2_ and H_2_S. 5 mL of 0.4% thymolphthalein pH indicator was added into 1 L NaOH solution to check the end point of absorption capacity. Finally, gas (primarily methane) went to back of the respective flow cell unit to measure the gas volume. Each experiment was done in triplicate along with the blank. Methane production was monitored continuously with the connected computer. Experiments were stopped when the cumulative gas production reached in the plateau. The BMP value is reported as the volume of methane produced per gram of VS added into the reactor.

### 2.6 Theoretical BMP

Theoretical BMP (BMP_th_) was calculated considering the COD value as well as elemental composition of substrate to compare with the experimental BMP. In this case it was considered that all the organic matter was degraded. The measured BMP values were compared with the BMP_th_ calculated by elemental composition. Finally, biodegradability was determined by measuring the VS after completing BMP measurements. Theoretical maximum BMP can be calculated from VS and COD of the substrate using Eq. (2) adapted from Ref. [33] as:

(2) $${BMP}_{th\;\left(COD\right)}=\frac{COD*\;RT}{{64\;PVS}_{added}}mL/g-VS$$

where BMP_thCOD_ is theoretical BMP, R is gas constant (R = 0.082 atm L/mol K), T is temperature of the reactor (K), P is atmospheric pressure (1 atm), VS_added_ (g) are the volatile solids of the substrate and nCH_4_ is number of moles of methane.

Based on the atomic composition (C, H, O, N) of substrate and considering the stoichiometric equation (Eq. (3)) below, theoretical BMP can be calculated using the Boyles equation (Eq. (4)).

(3) $$C_{n}H_{a}O_{b}N_{c}+{\left(n-\frac{a}{4}-\frac{b}{2}+\frac{3c}{4}\right)H}_{2}O=\left(\frac{n}{2}+\frac{a}{8}-\frac{b}{4}-\frac{3c}{8}\right){CH}_{4}+{\left(\frac{n}{2}-\frac{a}{8}+\frac{b}{4}+\frac{3c}{8}\right)CO}_{2}+c{NH}_{3}$$

(4) $${BMP}_{th\;\left(AtC\right)}=\frac{\left(\frac{n}{2}+\frac{a}{8}-\frac{b}{4}-\frac{3c}{8}\right)\times 22.4}{12n+a+16b+14c}$$

### 2.7 Kinetics of BMP reaction

The measured BMP data may be described by using an appropriate mathematical model. Variety of models have been reported to describe BMP data such as first order, first order with variable time dependency, combination of two first order, Monod type, Quadratic Monod type, Modified Gompertz kinetics [34,35]. Preliminary investigations were carried out for these different models and measured BMP data. These preliminary investigations indicated that the measured BMP data is adequately described by the combination of two first order (two stage) models as shown in Eq. (5):

(5) $$G=G_{1}\;\left(1-e^{-k_{1}t}\right)+G_{2}\;\left(1-e^{-k_{2}t}\right)$$

where G is the BMP at time t (day), k_1_ and k_2_ are rate constants. The maximum gas generation, G_max_ is the maximum BMP (mL/g-VS) and for two stage model used here, G_max_ = (G_1_ + G_2_). The results are discussed in the following.

## 3 Results and discussions

### 3.1 Rheological behaviour of untreated and treated sludge

**Fig. 2:**
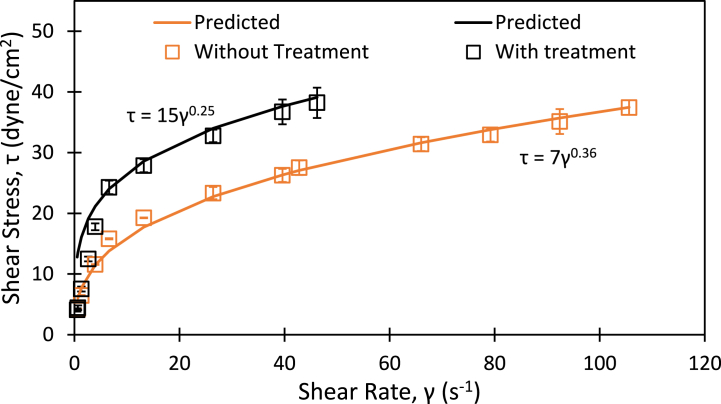
Flow curves of untreated and treated brown sludge. (For interpretation of the references to colour in this figure legend, the reader is referred to the Web version of this article.)

The HC treatment involves pumping of sludge through HC device. It is therefore important to understand influence of HC treatment on flow or rheological behaviour of brown sludge. The shear stress as a function of shear rate was therefore measured using the Brookfield rheometer. The measured dependence of shear stress with shear rate at the room temperature (25 °C) for treated and untreated brown sludge is showed in Fig. 2. It can be seen that brown sludge exhibits non-Newtonian (shear thinning) behaviour and stress versus strain data can be adequately described by a power law viscosity model. The HC treatment was found to increase consistency or viscosity. This may be attributed to the change in sludge structure with the HC treatment. HC treatment disintegrates solids and thereby may increase the viscosity. This is in agreement with the results reported by Ref. [36]. Other studies also reported that HC and shear stress degrade the sludge structure resulting reduction of particle size [37,38], smaller particles were likely to absorb moisture [30] and hence increased the sludge viscosity [39,40].

### 3.2 Effect of hydrodynamic cavitation treatment

Hydrodynamic cavitation pretreatment of brown sludge samples has been done for anaerobic digestion. The two main parameters we have considered for evaluation of solid degradation. Pressure drop across the HC device and number of passes through the HC device. We considered 250 kPa of pressure drop and investigated influence of 10, 20, 40 and 80 number of passes on biomethane yield.

**Fig. 3:**
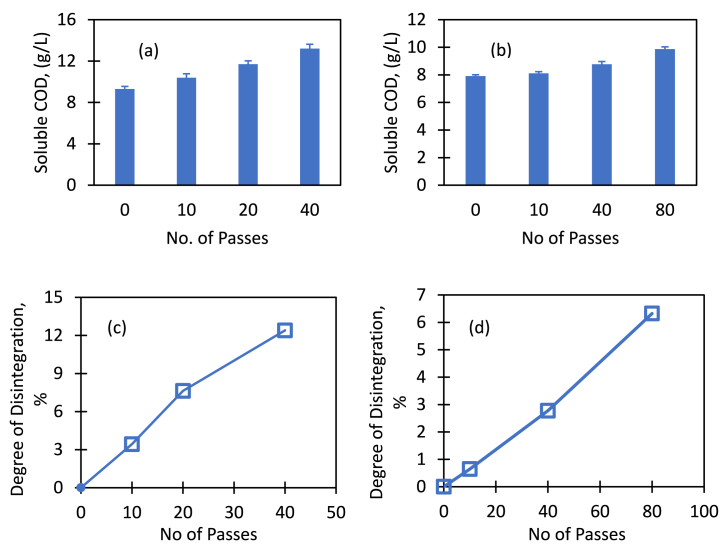
Soluble COD and degree of disintegration (DD) with the treatment severity.

HC treatment increased the solubility of substrate with the severity of treatment and hence the bioavailability in the sludge increased for fermentation reaction. Measured values of soluble COD (sCOD) and degree of disintegration (DD) with the treatment severity are shown in Fig. 3. It can be seen that sCOD increased with the number of passes indicating higher DD with the number of passes in the pretreatment process. sCOD of untreated brown sludge (sample 1) was 9.3 g/L and increased to 10.4 g/L, 11.7 g/L and 13.2 g/L when it was treated via hydrodynamic cavitation with 10, 20 and 40 number of passes (Fig. 3a). With 40 passes through HC device, there was nearly 42% increase in sCOD. Correspondingly DD was increased by 3.4%, 7.6% and 12.4% with number of passes as 10, 20 and 40 respectively (Fig. 3c). For the second sample of brown sludge (sample 2), sCOD increased 24.8%, from 7.9 g/L to 9.9 g/L (Fig. 3b) with 80 passes in HC treatment when the degree of disintegration had increased by 6.3% (Fig. 3d).

Yao et al., 2022 [30] reported sCOD increased from 50 mg/L to 320 mg/L and degree of disintegration was increased up to 22.98% with the 240 min of HC treatment of waste activated sludge using orifice with 20 holes of 2 mm each diameter as HC device. Other studies also reported using sludge from sewage treatment HC increased DD 12.7% by 20 passes of rotational cavitation generator [41], 10.1–26% using serrated discs-rotational generator HC [42] and 3.3–7.7% using pin discs-rotational generator HC [21]. In our study, we used vortex-based HC treatment. Compared to other cavitation devices such as orifice, venturi or rotor-stator for HC treatment of dairy sludge, vortex based HC devices have significantly lower erosion and early inception of cavitation and therefore offer effective pre-treatment at lower pressure drop (and therefore lower energy consumption) than other competing HC devices [43].

### 3.3 Morphology with the HC treatment

**Fig. 4:**
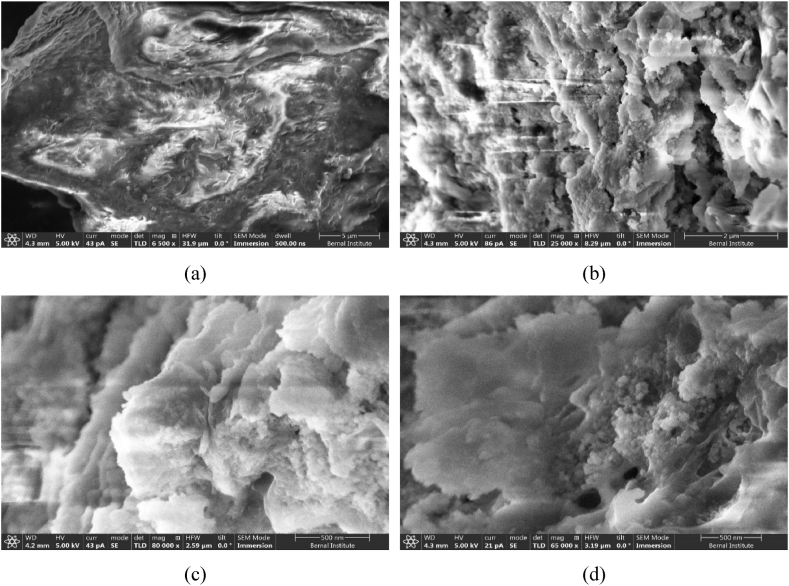
SEM images of the brown sludge (a) untreated and HC treated with (b) 10 passes, (c) 20 passes and (d) 40 number of passes.

Morphology changes with the severity of hydrodynamic cavitation treatment of substrate was observed in scanning electron microscope (SEM). Fig. 4a shows the untreated brown sludge image and Fig. 4b–4d show the structural changes of sludge with the HC treatment with the 10, 20 and 40 number of passes. The intense shear, shock waves and hydroxyl radicals generated by collapsing cavities break solid particles in brown sludge and enhance solid-liquid mass transfer leading to significant enhancement in solubilised COD (sCOD). The particle breakage and dissolution changes particle morphology, making it easier for microbes to degrade and convert the substrate to biomethane [28]. Other studies also reported similar findings on changes in particle morphology, enhancement of solid-liquid interfacial area and soluble COD with the HC treatment of grass silage, sugarcane bagasse, microcrystalline cellulose and activated sludges [31,43,44].

### 3.4 Biomethane production with the HC treatment of substrate

**Fig. 5:**
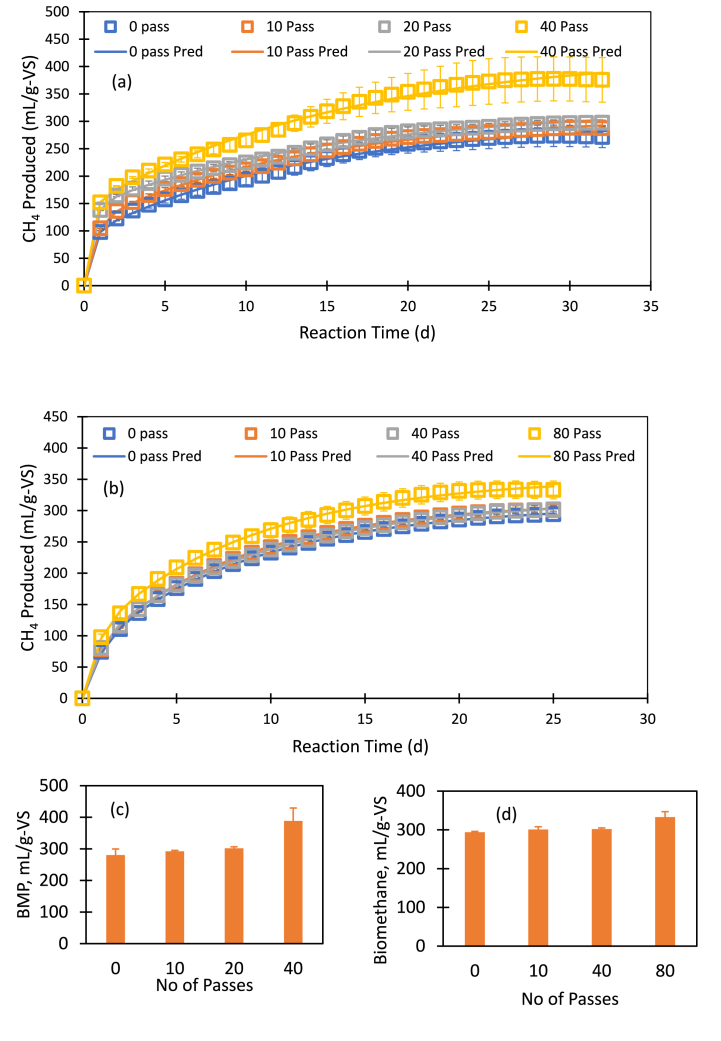
BMP with the severity of hydrodynamic cavitation pretreatment of brown sludge. (For interpretation of the references to colour in this figure legend, the reader is referred to the Web version of this article.)

Theoretical BMP calculated using CHNSO were 484.29 mL/g-VS and 403.9 mL/g-VS for two brown sludge samples. Fig. 5. Shows the BMP with the severity of hydrodynamic cavitation pretreatment of brown sludge. Maximum methane production using untreated brown sludge was 271 mL/g-VS using sample 1. While the HC treated sample using 10, 20 and 40 passes 287 mL, 297 mL and 376 mL of methane were produced respectively from each gram of VS (Fig. 5a). This sludge sample was produced 58% of theoretical BMP without treatment. HC treatment increased the methane production to 5.6, 9.6 and 38.7% while 10, 20 and 40 passed treated substrate were used in the reactor (Fig. 5c). It can be seen that enhancement in BMP is rather modest up to 20 passes through HC device (10% or less). However, with 40 passes, significant enhancement in BMP, up to 38.7% was observed. With 40 passes, the measured BMP crossed 80% of theoretical BMP (80.2%). Compared to this, Petkovšek et al., 2015 [41] reported that 20 passes through rotor-stator type of HC device increased soluble COD and hence methane yield in anaerobic digestion by 13% for treated waste activated sludge. Other previous studies reported the methane production of 473 mL/g-VS from polymer dewatered FOG waste [45], 344 mL/g-VS from co-digestion of 50% grease trap sludge and 50% waste activated sludge [46] and 339 mL/g-VS using primary and thickened activated sludge [47].

In case of 2nd types of brown sludge sample, the maximum methane production using untreated sludge was 294 mL/g-VS. While the HC treated sludge produced 301 mL, 302 mL and 333 mL of methane from each gram of VS using HC pretreated sample with the number of passes of 10, 40 and 80 respectively (Fig. 5b). HC treatment increased the methane production increased to 2.3, 2.7 and 13.3% with 10, 40 and 80 passes through HC device (Fig. 5d). BMP increased by 13.3% after the HC treatment of sludge using 80 passes which is much smaller than the increase observed with the first case. However, it is important to note that the obtained BMP after the HC treatment reached more than 80% of theoretical BMP (82.4%) which is quite similar to the observed ratio BMP to theoretical BMP for the first case. In the second case, the BMP of untreated sludge was rather high, nearly 73% of BMP_th_ compared to 58% of BMP_th_ observed for the first case. The HC treatment was able to push the BMP for both the sludge samples to more than 80% of BMP_th_ irrespective of the nature of untreated sludge sample.

### 3.5 Kinetics of BMP reaction

Anaerobic digestion is a complex and multistep process including hydrolysis, acidogenesis, acetogenesis and methanogenesis [48,49]. Kinetics of AD has been described by various models from simple rate limiting to complex steady state to dynamic models [34,35]. Kinetic models of first order, first order with variable time dependency, combination of two first order, Monod type, Quadratic Monod type, Modified Gompertz were tested to fit the experimental results of methane production. Best suited kinetic model to produce biomethane was found to be a combination of two first order kinetics (Eq. (5)). The measured BMP profiles for two samples were used to obtain parameters of Equation (5). The two-step kinetic model was found to describe the measured BMP data reasonably well (Fig. 5). The obtained values of model parameters for each HC treatment conditions for both the samples are listed in Table 2. It can be seen that the BMP of HC treated sludge, G_max_ (G_1_ + G_2_) increased from 300 to 426 mL/g-VS and 309–353 mL/g-VS while used 1st and 2nd types of brown sludge samples respectively. The two-step kinetic model will be useful for designing AD reactor based on brown sludge.

**Table 2:** Fitted parameters of dual first order kinetics model for brown sludge.

Parameters	Sample-1				Sample-2			
No. of Passes in HC	0	10	20	40	0	20	40	80
G_1_ (mL CH_4_/g-VS)	90.83	111.51	141.56	142.11	70.47	72.23	81.78	92.33
k_1_ (d^−1^)	2.52	1.67	2.26	3.03	1.06	1.20	1.08	1.31
G_2_ (mL CH_4_/g-VS)	209.14	198.83	182.89	283.94	236.76	244.53	237.33	161.08
k_2_ (d^−1^)	0.07	0.07	0.07	0.06	0.11	0.12	1.11	0.11
SumE	649	278	411	1323	17	30	23	195
G_max_ (mL CH_4_/g-VS)	299.97	310.34	324.45	426.05	308.99	315.01	319.11	353.42

### 3.6 Influence of HC treatment on VS conversion

**Fig. 6:**
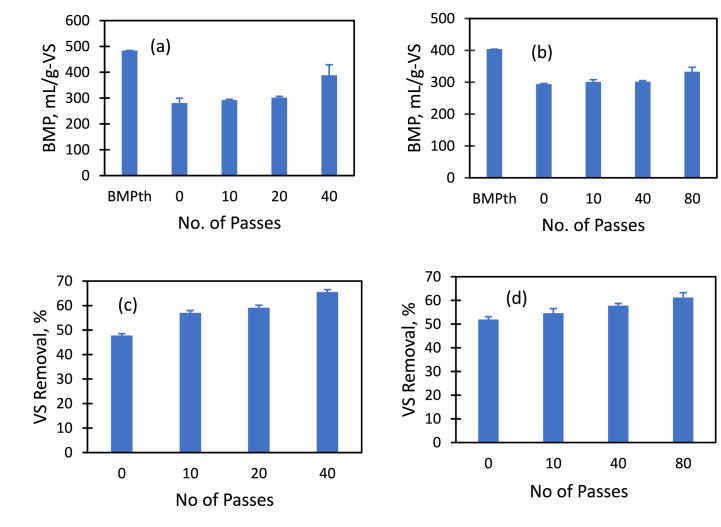
BMP and VS removal with and without HC treatment.

Influence of HC treatment of brown sludge on VS conversion was quantified by characterising VS before and after the end of BMP measurements. The results of BMP and VS removal at the end of BMP measurements are shown in Fig. 6. It can be seen that VS removal was increased from 48% for untreated sludge to 65.5% for sludge with 40 passes through HC device (Fig. 6c). This led to corresponding increased the BMP from 280.58 mL/g-VS to 388.35 mL/g-VS when treated the brown sludge with 40 passes (Fig. 6a). Higher VS conversion was due to the enhanced solubilisation of COD and better accessibility of digestible matter after HC treatment. Similarly, it has been observed that BMP increased from 294 mL/g-VS to 333 mL/g-VS when the second sample of sludge was HC treated with 80 number of passes (Fig. 6b). In that case, VS reduction was 51.93% for untreated sample and 61.24% for the treated substrate (Fig. 6d).

**Fig. 7:**
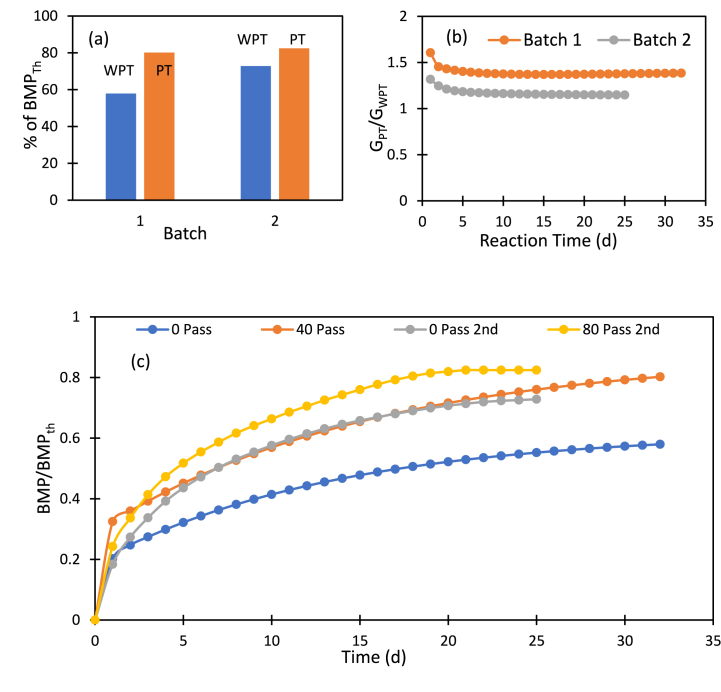
Comparison of produced BMP using two types of brown sludge samples. (For interpretation of the references to colour in this figure legend, the reader is referred to the Web version of this article.)

Prediction of biomethane potential using empirical formula is widely accepted though equations assume that all the organic matter is biodegradable [50,51]. Fig. 7(a) shows the percentage of theoretical BMP reached in the anaerobic digestion reaction using untreated and HC treated sample which produced highest yields. Sludge sample-1 reached maximum 58% and 80% of BMP_th_ with untreated and treated sludge respectively while 73% and 82% of BMP_th_ for sludge sample-2. These results indicate that HC treatment improved the methane production more when the ratio BMP_th_/BMP of untreated sample is higher (Fig. 7c). This ratio was 1.6 for sample-1 and 1.3 for sample-2 (Fig. 7b). With vortex based HC treatment presented in this work, this ratio was brought down to less than 1.25 for both types of brown sludge samples. This observation will be useful to evaluate commercial feasibility of the HC based treatment for brown sludge.

### 3.7 Technoeconomic analysis of biomethane production from brown sludge

Vortex based HC treatment of brown sludge enhances the biomethane production. Based on the results obtained this cavitation pretreatment can be scaled up for industrial application. It is necessary to calculate energy and economics in HC treatment of brown sludge to increase the biomethane yield. In HC treatment, cost of energy is required only to pump the sludge through cavitation device which is proportional to the number passes and pressure drop required since this device does not require any chemicals. The required energy for pretreatment of a ton of brown sludge can be calculated using Eq. (6).

(6) $$E_{pretreatment}=\frac{\Delta P\;n}{3.6\;x\;10^{6}\eta }\frac{kWh}{ton\;of\;sludge}$$

ΔP is the pressure drop (kW), n is the number of passes and η is the efficiency of pump used. Because of the pretreatment bioavailability of sludge increased and hence the biomethane yields also increased. Energy gain can be calculated by subtracting the energy produced using pretreated sludge and untreated sludge (Eq. (7)).

(7) $$E_{gained}=\Delta H_{cal}y_{VS}\;{\left[\Delta G_{\max}\right]}_{\left(pretreated-untreated\right)}\frac{kWh}{m^{3}of\;sludge}$$

where ΔH_cal_ is the electricity potential of methane (10 kWh/m^3^), y_vs_ is the ton of VS in each m^3^ of sludge ΔG_max_ is the methane yield increased (m^3^/ton-VS). Energy required for HC treatment was 2.8 kWh/m^3^-sludge and energy gained in the form of methane was 42.6 kWh/m^3^-sludge. The energy required for HC treatment here is much lower than the other published results reported for cattle manure 440–660 kWh/ton-TS and for silage 280 kWh/ton-TS [52,53]. Considering the 1st sample with the 40 passed HC treatment containing 0.036 ton-VS/m^3^-sludge, methane yield increased from 271 to 376 m^3^/ton-VS and net energy gained will be 37.8 kWh/m^3^-sludge. Technoeconomic analysis may be expanded to include additional parameters such as capital investment, operational costs, and potential revenue streams to provide a more comprehensive assessment. The results presented in this work clearly indicate the potential of HC pretreatment of brown sludge followed by biomethane production via anaerobic digestion for significantly enhancing dairy sludge valorisation.

## 4 Conclusions

In this study, we investigated influence of hydrodynamic cavitation treatment of brown sludge from wastewater treatment plant of dairy processing industry. Vortex based HC device was used. Brown sludge exhibited non-Newtonian behaviour (shear thinning) and HC treatment increased effective viscosity of brown sludge. HC treatment increased the soluble COD and the degree of disintegration and hence increased the bioavailability of digestible substrate for anaerobic digestion. Observed enhancement in BMP with HC treatment increased with increase in number of passes. The extent of performance enhancement with pre-treatment is higher if the difference in BMP_Th_ and BMP without treatment was larger. With adequate number of passes, vortex based HC treatment was shown to be able to push BMP higher than 80% of BMP_Th_. VS removal was found to be more than 65% after HC treatment. The BMP data was well described by the two step (first order) kinetics model. Technoeconomic analysis showed that, the energy gained was 37.8 kWh/m^3^-sludge with HC treatment of brown sludge followed by biomethane production. The results will be useful for harnessing HC treatment for enhancing valorisation of brown sludge from dairy industry.

Further research is needed to address the challenges associated with different types of sludge and seasonal as well as industry wide variations in sludge composition. This will help determine the applicability and potential benefits of HC treatment. Long-term studies using continuous anaerobic reactors are necessary to assess the stability and consistency of enhanced methane production achieved through HC treatment, ensuring its sustainability and reliability.

## Author contribution statement

Md Saiful Islam: Performed the experiments; Analyzed and interpreted the data; Contributed reagents, materials, analysis tools or data; Wrote the paper.

Vivek V. Ranade: Conceived and designed the experiments; Analyzed and interpreted the data; Contributed reagents, materials, analysis tools or data; Wrote the paper.

## Data availability statement

Data will be made available on request.

## Authorship statements

**Md Saiful Islam:** Conceptualization, Methodology and Experiment, Data Processing, Writing Manuscript.

**Vivek V. Ranade:** Supervision, Project Administration, Funding Acquisition and reviewing.

## Declaration of competing interest

The authors declare that they have no known competing financial interests or personal relationships that could have appeared to influence the work reported in this paper
